# Editorial: Benign paroxysmal positional vertigo

**DOI:** 10.3389/fneur.2024.1358860

**Published:** 2024-02-13

**Authors:** Anita Bhandari, Shivam Sharma, Francisco Carlos Zuma E. Maia, Bernardo Faria Ramos, Eduardo Martin-Sanz

**Affiliations:** ^1^NeuroEquilibrium Diagnostic Systems Pvt. Ltd., Jaipur, India; ^2^Monilek Hospital and Research Center, Jaipur, Rajasthan, India; ^3^Department of Otorhinolaryngology and INSCER, Pontifical Catholic University of Rio Grande do Sul, Porto Alegre, Brazil; ^4^Department of Otorhinolaryngology, Federal University of Espirito Santo, Vitoria, Brazil; ^5^European University of Madrid, Villaviciosa de Odón, Spain

**Keywords:** BPPV, maneuver, simulation, vestibular tests, mechanical chair

Recent strides made in the understanding and management of benign paroxysmal positional vertigo (BPPV) represent a transformative era in vestibular medicine. This vestibular disorder, characterized by brief episodes of severe vertigo triggered by specific head movements, has long been a focus of research and clinical attention. Over the past few years, a confluence of advancements in diagnostics, treatment modalities, and molecular insights has propelled the field forward, fostering a more nuanced comprehension of BPPV and offering innovative approaches to its management (Hu et al.). The character of the nystagmus generated on positional tests is considered the primary diagnostic parameter to determine the presence of BPPV and to decide which canal is affected. Simulation models demonstrating the movement of otolith in the three-dimensional labyrinth have furthered our understanding of BPPV and its treatment (1, 2). It has been seen that the sequence of performing the positional tests can affect the test outcome (2, 3). An important development in the diagnostic landscape of BPPV is the proposal of additional positional tests alongside the traditional supine roll test. The Upright BPPV Protocol for horizontal canal BPPV was described to give minimum stimulation to decide the side of BPPV (4). However, it has undergone a gradual implementation by experienced vertigo clinicians as an additional examination for any BPPV subtype. In addition, a 45° tilt of the head has been proposed to explore the presence of BPPV affecting posterior and anterior canals to increase the sensitivity of the bow and lean test in the diagnosis of vertical canal BPPV (5). The 60° roll test and prone roll test are supplementary tests that have also been proposed to aid in determining the position of otoliths within the affected canals and in distinguishing between canalolithiasis and cupulolithiasis (6).

Canalith repositioning procedures (CRPs) play a pivotal role in the evolving landscape of BPPV management, particularly for single-canal BPPV (7, 8). A shortened forced position maneuver where the patient is kept in the lateral position on the healthy side in hc-BPPV has been proposed as an alternate to the well-established prolonged forced positioning maneuver (9). The efficacy of these maneuvers lies in their ability to swiftly alleviate symptoms by repositioning displaced otoliths from the affected canals. While the success of CRPs in single-canal BPPV is well established, the scenario becomes more complicated when dealing with multiple-canal involvement. The challenges in managing multiple-canal BPPV include the need for repeated treatments and a heightened risk of recurrence (Alfarghal et al.).

One notable revelation from recent studies is the efficiency and safety of the mechanical rotatory chair (MRC) in treating posterior BPPV (Zhang and Zhu). The MRC, using Semont, Epley, or 360-degree backwards somersault maneuver, has demonstrated efficacy, with all maneuvers being equally effective (Hougaard et al.). This finding positions the MRC as a valuable addition to the existing repertoire of BPPV treatments, offering both effectiveness and safety in clinical practice. The MRC records nystagmus during positional tests and repositioning maneuvers, allowing the clinician to effectively follow the otolith movement.

Recurrence of BPPV is a challenging clinical scenario. It has been seen that patients with abnormal vestibular function are at a higher risk of incidence of BPPV and its recurrence. Vestibular disorders such as vestibular migraine, Meniere's disease, labyrinthitis, and labyrinthine concussion, etc., are more prone to recurrence, stressing the importance of vestibular tests in BPPV patients (Zhang et al.). The identification of endolymphatic hydrops and canal paresis as independent risk factors for short-term recurrent BPPV represents a newfound knowledge, allowing for a more targeted approach to treatment (Zhang and Zhu). Bioinformatics analyses have delved into the molecular basis of BPPV, shedding light on the intricate connections between oxidative stress, extracellular matrix degradation, and immune-mediated inflammatory responses. The identification of these molecular factors contributing to recurrent BPPV, especially in younger patients, opens up avenues for targeted therapeutic interventions. This not only represents a significant leap forward in our understanding of the disorder but also lays the foundation for more personalized and effective treatments.

Future directions in BPPV research are now charting a course toward the refinement of individualized treatments. This involves a specific focus on the recognition of precise otolith locations through the identification of characteristic nystagmus patterns and optimum repositioning maneuvers and addressing residual symptoms. Additionally, there is a growing emphasis on unraveling the intricacies of secondary inner ear diseases and their interplay with BPPV. This comprehensive approach aims to address not only the primary symptoms but also the broader spectrum of factors influencing the course and prognosis of BPPV. The holistic consideration of these risk factors enables clinicians to tailor treatment plans to individual patient needs, thereby fostering more effective and sustainable outcomes. As research continues to unfold, the collaborative efforts of clinicians and researchers will undoubtedly play a pivotal role in further refining BPPV management, paving the way for a future where the complexities of this disorder are met with precision and tailored solutions.

## Author contributions

AB: Formal analysis, Supervision, Writing—original draft, Writing—review & editing. SS: Writing—original draft, Writing—review & editing. FZ: Writing—original draft, Writing—review & editing. BR: Writing—original draft, Writing—review & editing. EM-S: Writing—original draft, Writing—review & editing.
